# A drug–drug interaction study of a novel, selective urate reabsorption inhibitor dotinurad and the non-steroidal anti-inflammatory drug oxaprozin in healthy adult males

**DOI:** 10.1007/s10157-020-01855-2

**Published:** 2020-02-19

**Authors:** Kenichi Furihata, Katsuaki Nagasawa, Atsushi Hagino, Yuji Kumagai

**Affiliations:** 1 P-One Clinic, Keikokai Medical Corporation, 8-1 Yokamachi, Hachioji, Tokyo 192-0071 Japan; 2 Clinical Research Department, Mochida Pharmaceutical Co., Ltd., 1-22 Yotsuya, Shinjuku-ku, Tokyo, 160-0004 Japan; 3 Kitasato University School of Medicine, 1-15-1 Kitasato, Minami-ku, Sagamihara, Kanagawa 252-0374 Japan

**Keywords:** FYU-981, Dotinurad, Urate transporter 1 (URAT1), Selective urate reabsorption inhibitor (SURI), Pharmacokinetics, Drug–drug interaction

## Abstract

**Background:**

Dotinurad is a novel, selective urate reabsorption inhibitor, which reduces serum uric acid levels by inhibiting the urate transporter 1. The results of nonclinical studies indicated the possibility that the concomitant use of the non-steroidal anti-inflammatory drug oxaprozin affects the pharmacokinetics of dotinurad. We evaluated drug–drug interactions with respect to the pharmacokinetics and safety of dotinurad when co-administered with oxaprozin.

**Methods:**

This was an open-label, two-period, add-on study in healthy adult males. For a single dose of 4 mg of dotinurad with and without oxaprozin, we compared its pharmacokinetic parameters and evaluated safety.

**Results:**

This study enrolled 12 subjects, 11 of whom completed the study. The geometric mean ratio (90% confidence interval [CI]) of the urinary excretion rate of glucuronate conjugates of dotinurad after co-administration with oxaprozin compared to administration of dotinurad alone was 0.657 (0.624–0.692), while the geometric mean ratios (90% CIs) of the maximum plasma concentration and area under the plasma concentration–time curve from time zero to infinity (AUC_0–inf_) were 0.982 (0.945–1.021) and 1.165 (1.114–1.219), respectively. During the study, two adverse events occurred after administration of dotinurad alone and one occurred after administration of oxaprozin alone.

**Conclusions:**

In comparison with administration of dotinurad alone, co-administration with oxaprozin was associated with a 34.3% decrease in the urinary excretion rate of the glucuronate conjugates of dotinurad, and a 16.5% increase in AUC_0–inf_ of dotinurad. However, no clinically meaningful drug–drug interactions were observed. Administration of dotinurad alone was similar safety to co-administration with oxaprozin.

**Clinical trial registration:**

ClinicalTrials.gov Identifier: NCT03350386.

## Introduction

The numbers of patients with hyperuricemia and those with gout are increasing in Japan, with the prevalence of hyperuricemia in males in their 30s reaching 30% [1]. Hyperuricemia and gout are recently being considered as lifestyle-related diseases along with hypertension, dyslipidemia, and diabetes mellitus, and the Japanese Society of Gout and Nucleic Acid Metabolism has issued management guidelines [1, 2]. Correcting the hyperuricemic condition and appropriately controlling uric acid levels are critical for the prevention of acute gouty arthritis, gouty nephropathy, and urinary calculi and may also protect renal function and reduce cardiovascular risks in hyperuricemic patients [3, 4].

Hyperuricemia is caused by uric acid overproduction, uric acid underexcretion, or their combination. The Japanese guideline for the management of hyperuricemia and gout recommends that uricostatic drugs be used in overproduction-type patients and uricosuric drugs be used in underexcretion-type patients [1]. Uricostatic drugs include xanthine oxidoreductase inhibitors (XOIs) such as allopurinol, febuxostat, and topiroxostat, and uricosuric drugs include probenecid and benzbromarone. The uricosuric drug lesinurad was recently approved in the US and EU as an add-on therapy to an XOI [5, 6]. Lesinurad requires careful administration when combined with a cytochrome P450 (CYP) 2C9 inhibitor (e.g., fluconazole, amiodarone) and in CYP2C9 poor metabolizers because lesinurad undergoes oxidative metabolism by CYP2C9. It was reported that lesinurad, which also acts as a CYP3A4 inducer, reduces plasma concentration of a CYP3A4 substrate when used concomitantly [6]. Benzbromarone, a CYP2C9 inhibitor, should be administered carefully when combined with a coumarin anticoagulant (e.g., warfarin), which CYP2C9 metabolizes [7]. The XOI febuxostat reportedly increases the area under the plasma concentration–time curve (AUC) of naproxen, a non-steroidal anti-inflammatory drug (NSAID), through its inhibition of UDP-glucuronosyltransferase (UGT) [8]. Under such circumstances, development of anti-hyperuricemic agents with fewer drug–drug interactions, particularly with uricosuric drugs, is expected.

Dotinurad (development code: FYU-981) selectively inhibits a urate exchanger, urate transporter 1 (URAT1), which reduces serum uric acid levels by stimulating urate excretion via inhibition of reabsorption of uric acid [9, 10]. Dotinurad is a potent URAT1 inhibitor with minimal effect on other urate secretion transporters, such as ATP-binding cassette subfamily G member 2 (ABCG2), organic anion transporter (OAT) 1, and OAT3, compared to commercially available uricosuric drugs, namely probenecid, benzbromarone, and lesinurad. A clinical study in subjects with renal dysfunction demonstrated that glucuronate conjugates and sulfate conjugates are the major metabolites of dotinurad and that they are excreted in urine [11]. A nonclinical study investigated the effects of drug–drug interactions in terms of metabolism and plasma protein binding between dotinurad and potential co-administered drugs (NSAIDs, XOIs, anti-hypertension drugs, antihyperlipidemic drugs, antidiabetic drugs, and others) [12]. An assessment of dotinurad’s glucuronidation by potentially co-administered drugs in human liver microsome revealed that the NSAID oxaprozin was the most potent inhibitor of glucuronidation. When human plasma protein-binding substitution activities were examined by ultrafiltration method, oxaprozin and naproxen were found to lower the plasma protein binding rate of dotinurad. In contrast, dotinurad had no effects on the plasma protein binding rates of potentially co-administered drugs nor on their CYPs, UGTs, sulfotransferases, and drug transporters at clinical doses in several in vitro studies [12]. As a result, oxaprozin and naproxen have the potential to increase the AUC of dotinurad by inhibiting its glucuronidation. They also have the potential to lower the plasma protein binding rate of dotinurad, which in turn facilitates its metabolism, leading to a decrease in its AUC. Based on the findings of in vitro studies suggesting that oxaprozin is most likely to interact with the pharmacokinetics (PK) of dotinurad, we conducted a drug–drug interaction study to evaluate the effects of concomitant oxaprozin on the PK and safety of dotinurad in healthy adult males.

## Methods

### Study subjects

Males aged 20 to 35 years with body mass index of 18.5 to 25.0 kg/m^2^ were eligible to participate in the study. Prospective subject’s health was assessed through medical history, physical examination, screening laboratory tests, standard 12-lead electrocardiography, and abdominal ultrasound. Those who met any of the following criteria were excluded: individuals with or with a history of any cardiac, hepatic, renal, pulmonary, hematological, gastrointestinal, thyroidal, neuropsychiatric, metabolic/electrolyte disorders; individuals with a peptic ulcer; individuals with a urinary calculus; individuals with drug allergy or drug hypersensitivity; individuals with symptoms of allergy (including hypersensitivity) to oxaprozin or with a history of allergy to oxaprozin; individuals with or with a history of aspirin-induced asthma (NSAID-induced asthmatic attacks); individuals with symptoms of drug abuse or alcoholism or with a history of drug abuse or alcoholism; individuals who smoked at least 11 cigarettes per day during the three months prior to screening or who were unable to refrain from smoking on the day of the study visit and during hospitalization; individuals who donated whole blood or blood components in an amount exceeding the defined amount during a given period of time; individuals who ingested St. John’s wort or foods and drinks containing St. John’s wort within four weeks of the first dose of dotinurad; individuals who ingested grapefruit or foods and drinks containing grapefruit within seven days of the first dose of the study drug or who used drugs (including over-the-counter drugs and drugs requiring guidance) or vitamins (including energy drinks) within seven days of the first dose of the study drug; and individuals who participated in another clinical study in the defined period of time.

### Study design

**Fig. 1:**
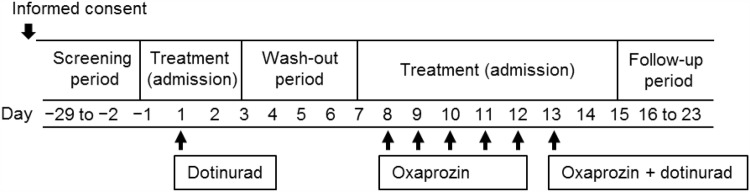
Study design

This was a single-center, open-label, two-period, sequential-group, add-on, drug–drug interaction study in healthy adult males conducted at a Japanese clinical pharmacology study center. Following a screening period ranging from 29 days before, to the day before the study drug administration (Day -1), eligible subjects were admitted to the study site on Day-1, received a single 4 mg oral dose of dotinurad in a fed state, and were discharged 48 h postdose. Having completed seven days of washout following dotinurad dosing, subjects who were considered eligible to remain in the study were admitted to the study site once more on Day 7, and received oxaprozin 600 mg oral dose in a fed state once daily for five days. On Day 13, they received dotinurad 4 mg and oxaprozin 600 mg in a fed state and were discharged 48 h after co-administration (Fig. 1). They underwent a follow-up examination between six and ten days after co-administration for safety evaluation.

### Sampling and treatments

Blood sampling for determination of plasma drug concentrations was performed at baseline (0 h) and 0.5, 1, 2, 4, 6, 8, 12, 24, 36, and 48 h after administration of dotinurad alone and after co-administration with oxaprozin. A blood sample was drawn, in the amount of 5 mL at each time point except for 10 mL at 4 h after drug administration, into a heparin-containing blood collection tube and centrifuged at 3000 rpm for 10 min at 4 °C. A plasma sample obtained at each time point was then stored at – 20 °C before shipping in a chilled condition with dry ice to Sekisui Medical Co., Ltd. (Ibaraki, Japan) for determination of plasma drug concentrations. Based on the results of nonclinical studies indicating a low potential for dotinurad to affect the PK of oxaprozin, plasma oxaprozin concentrations before co-administration and 24 h after co-administration were measured to check only whether plasma oxaprozin concentrations reach a steady state or not.

Urine samples for determination of dotinurad’s metabolites were collected at 0–6, 6–12, 12–24, and 24–48 h after dotinurad administration with and without oxaprozin. After the pH of the urine sample collected after each time interval was adjusted to ≤ 7.0, the sample was stored at – 20 °C before shipping in a chilled condition with dry ice to Fuji Yakuhin Co., Ltd. (Saitama, Japan) for determination of urinary concentrations of dotinurad’s metabolites.

### Bioanalytical methods

Plasma concentrations of dotinurad and oxaprozin were determined by a validated liquid chromatography-tandem mass spectrometry (LC-MS/MS) method (LC: LC-20AD system for dotinurad, SHIMADZU; LC-10ADvp, CTO-10ASvp, DGU-14A, and SIL-HTc for oxaprozin, SHIMADZU. MS/MS: API4000, SCIEX). The lower limit of quantification (LLOQ) was 1 ng/mL for dotinurad and 1 μg/mL for oxaprozin. Ultrafiltered plasma samples were used to determine unbound plasma concentrations of dotinurad by a validated LC–MS/MS method (LC:LC-20AD system, SHIMADZU. MS/MS: API4000, SCIEX). The LLOQ was 0.1 ng/mL.

Urinary concentrations of metabolites (glucuronate conjugates and sulfate conjugates) were determined by a validated LC-MS/MS method (LC: Nexera X2 and Prominence, SHIMADZU. MS/MS: Triple Quad 4500, SCIEX). The LLOQ was 10 ng/mL for both metabolites.

### Statistical analysis

The target number of subjects was determined to be 12 subjects in consideration of the feasibility of the study, without statistical calculation of a sample size. The statistical analysis plan was finalized prior to data lock.

The primary analysis was to evaluate changes in plasma dotinurad concentrations at individual time points after administration of dotinurad alone and after co-administration with oxaprozin, and their summary statistics. The PK parameters of dotinurad, namely maximum plasma concentration (*C*_max_), time to reach the peak plasma concentration (*T*_max_), elimination half-life (*T*_1/2_), AUC_0–inf_, total clearance/fraction of dose absorbed (CL_tot_/*F*), and distribution volume/fraction of dose absorbed (Vd/*F*), were calculated using the PK analysis software Phoenix WinNonlin Version 6.1 (CERTARA), and their summary statistics were evaluated after administration of dotinurad alone and after co-administration with oxaprozin.

The summary statistics of fraction of dose excreted in urine (*f*_e_) of dotinurad’s metabolites after administration of dotinurad alone and after co-administration with oxaprozin were also calculated. Furthermore, the geometric mean ratios, together with the two-sided 90% confidence intervals (CIs), of plasma dotinurad concentrations and the PK parameters of urinary metabolites of dotinurad when co-administered with oxaprozin compared to when administered alone were calculated for each subject. The summary statistics of unbound fraction of dotinurad in plasma at the time of *C*_max_ after administration of dotinurad alone and after co-administration with oxaprozin were calculated, and their geometric mean ratios, together with the two-sided 90% CIs, after co-administration of dotinurad and oxaprozin compared to administration of dotinurad alone were also calculated for each subject.

The PK analysis population included subjects who received the study drug and who had at least one postdose measurement of plasma dotinurad concentration.

Adverse events (AEs), a measure used for safety evaluation, were coded by preferred term using the Medical Dictionary for Regulatory Activities (MedDRA) version 20.1 (Japanese Maintenance Organization, Tokyo, Japan) and tabulated. The investigator observed/examined/assessed AEs, subjective symptoms and objective findings, vital signs, electrocardiography, and laboratory test values.

The safety analysis population included subjects who received at least one dose of dotinurad and who had data for safety evaluation.

## Results

### Subjects

**Fig. 2:**
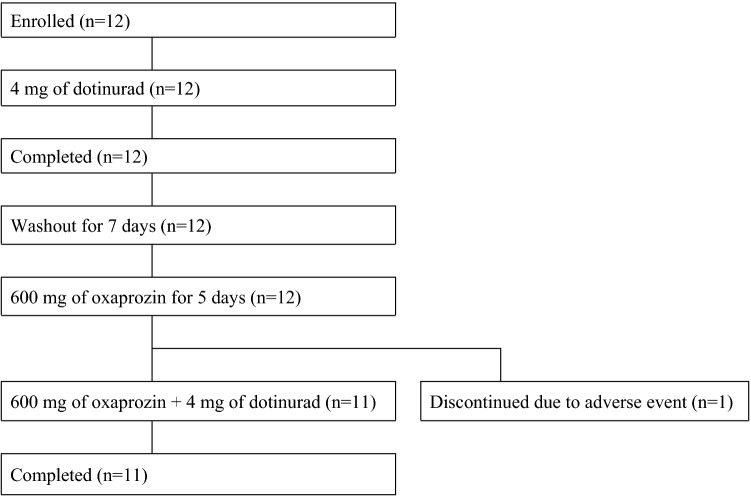
Subject disposition

A total of 12 subjects enrolled in the study and received dotinurad alone. One subject experienced an AE during repeated dosing of oxaprozin alone and was withdrawn from the study. The remaining 11 subjects completed the study (Fig. 2). Plasma dotinurad concentrations and urinary metabolites concentrations in 12 subjects who received treatment with dotinurad alone and 11 subjects who received treatment with oxaprozin and dotinurad were determined and their PK parameters were calculated. The safety analysis population included all 12 subjects who received the study drugs. Table 1 shows the demographic of subjects.

**Table 1:** Subject baseline demographics (*n* = 12)

Characteristic	Value
Age, years (range)	25.7 ± 5.4 (20–34)
Gender	
Male	12 (100.0)
Female	0 (0.0)
Height, cm	176.18 ± 4.88
Weight, kg	66.92 ± 6.76
Body mass index, kg/m^2^	21.53 ± 1.56
Race	
Asian	12 (100.0)
Others	0 (0.0)

Mean ± SD or *n* (%)

*SD* standard deviation

### Pharmacokinetic analysis

**Fig. 3:**
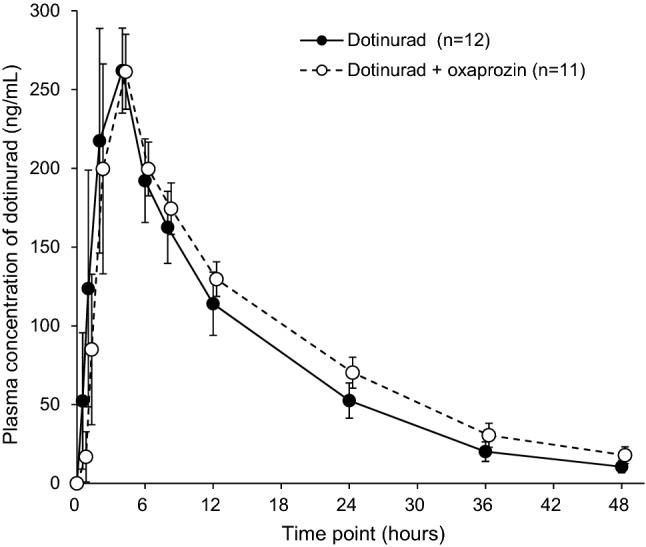
Mean (± SD) plasma concentration versus time point for dotinurad. *SD* standard deviation

**Fig. 4:**
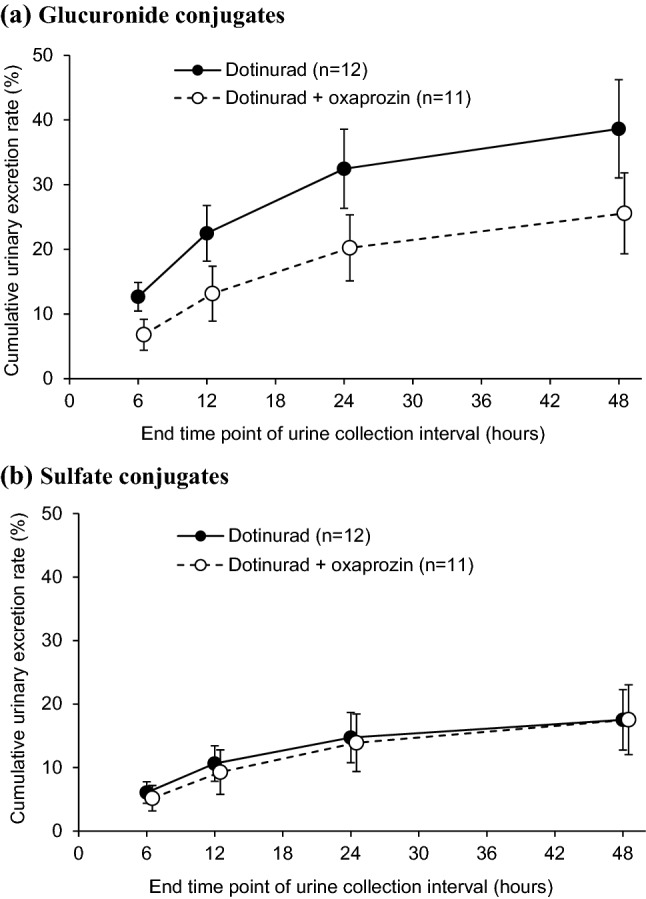
Mean (± SD) cumulative urinary excretion rate versus end time point of urine collection interval for metabolites of dotinurad. *SD* standard deviation

Figures 3 and 4 show time profiles in the mean plasma dotinurad concentrations and cumulative urinary excretion rates of metabolites, respectively, after administration of dotinurad alone and after co-administration with oxaprozin. Table 2 shows the summary statistics of PK parameters of dotinurad, urinary excretion rates of metabolites, and unbound fraction of dotinurad in plasma at the time of *C*_max_.

**Table 2:** Summary statistics and geometric mean ratios of pharmacokinetic parameters of dotinurad alone and with oxaprozin

Parameter	Dotinurad alone (*n* = 12)		Dotinurad and oxaprozin (*n* = 11)		Ratio^a^ (*n* = 11)
	Mean	SD	Mean	SD	Geometric mean ratio (90% CI)
*C*_max_ (ng/mL)	270.77	26.61	266.11	27.01	0.982 (0.945–1.021)
*T*_max_ (h)	3.67	0.78	3.64	0.81	1.000 (0.844–1.185)
*T*_1/2_ (h)	9.85	1.06	11.89	1.33	1.205 (1.176–1.236)
AUC_0–inf_ (ng・h/mL)	3846	579	4487	480	1.165 (1.114–1.219)
CL_tot_/*F* (L/h)	1.065	0.184	0.901	0.099	0.858 (0.820–0.898)
Vd/*F* (L)	14.93	1.48	15.30	0.94	1.035 (0.999–1.071)
*f*_e_ of glucuronide conjugates (%)	38.63	7.59	25.57	6.25	0.657 (0.624–0.692)
*f*_e_ of sulfonate conjugates (%)	17.52	4.75	17.53	5.49	0.968 (0.900–1.041)
Unbound fraction in plasma at *C*_max_ (%)	0.534	0.067	0.859	0.100	1.615 (1.454–1.794)

*AUC*_*0–inf*_ area under the plasma concentration–time curve from time zero to infinity, *CI* confidence interval, *CL*_*tot*_*/F* total clearance/fraction of dose absorbed, *C*_*max*_ maximum plasma concentration, *f*_*e*_ fraction of dose excreted in urine, *SD* standard deviation, *T*_*1/2*_ elimination half-life, *T*_*max*_ time to reach the peak plasma concentration, *Vd/F* distribution volume/fraction of dose absorbed

^a^Ratio was calculated as follows. [Dotinurad and oxaprozin]/[Dotinurad alone] for each subject

The geometric mean ratios (90% CIs) of *C*_max_, *T*_max_, and Vd/*F* of dotinurad after co-administration compared to administration of dotinurad alone were 0.982 (0.945–1.021), 1.000 (0.844–1.185), and 1.035 (0.999–1.071), respectively, showing no differences between dotinurad alone and with oxaprozin. The geometric mean ratios (90% CIs) of AUC_0–inf_, *T*_1/2_, and CL_tot_/*F* were 1.165 (1.114–1.219), 1.205 (1.176–1.236), and 0.858 (0.820–0.898), respectively, demonstrating increased AUC_0–inf_, prolonged *T*_1/2_, and decreased CL_tot_/*F* of dotinurad after co-administration with oxaprozin.

The geometric mean ratios (90% CIs) of *f*_e_ of glucuronate conjugates and sulfate conjugates after co-administration with oxaprozin compared to administration of dotinurad alone were 0.657 (0.624–0.692) and 0.968 (0.900–1.041), respectively. The geometric mean ratio (90% CI) of unbound fraction of dotinurad in plasma at the time of *C*_max_ after co-administration with oxaprozin compared to administration of dotinurad alone was 1.615 (1.454–1.794). These results demonstrated that co-administration with oxaprozin reduced the urinary excretion rate of glucuronate conjugates and increased unbound fraction of dotinurad in plasma.

### Safety analysis

Table 3 summarizes AEs reported during the study. Two of 12 subjects experienced three AEs, for which a causal relationship with dotinurad and oxaprozin was ruled out. Two events occurred in two subjects after treatment with dotinurad alone and one event occurred in one subject after treatment with oxaprozin alone. No AEs were reported after co-administration. One event of pharyngitis on Day 6 of dotinurad alone and one event of peritonsillar abscess on Day 12 of oxaprozin alone, both of which occurred in the same subject, resulted in study discontinuation. The AE of peritonsillar abscess was serious and resolved after treatment at another medical facility. One event involving an increase in the urinary beta 2 microglobulin level was reported as a mild AE in one subject. Other than this, no clinically meaningful changes in laboratory test values, vital signs, or electrocardiograms were observed during the study.

**Table 3:** Adverse events

Preferred term	Dotinurad alone (*n* = 12)		Oxaprozin alone (*n* = 12)		Dotinurad and oxaprozin (*n* = 11)	
	Number of events	Incidence (%)	Number of events	Incidence (%)	Number of events	Incidence (%)
Peritonsillar abscess	0	0.0	1	8.3	0	0.0
Pharyngitis	1	8.3	0	0.0	0	0.0
Urine beta 2 microglobulin increased	1	8.3	0	0.0	0	0.0

Preferred terms specified by MedDRA version 20.1

## Discussion

This study evaluated the effects and safety of co-administration of dotinurad and oxaprozin in healthy adult males.

The plasma oxaprozin concentrations (mean ± standard deviation [SD]) before co-administration with dotinurad (i.e., 24 h after repeated dosing of oxaprozin for five days) and 24 h after co-administration were 102.30 ± 10.45 μg/mL (*n* = 11) and 102.18 ± 10.39 μg/mL (*n* = 11), respectively, showing similarity between before and after co-administration. These values are comparable to those reported by Knuth et al. [13], implying that the effect of dotinurad on oxaprozin’s PK is insignificant. The fact that oxaprozin concentration reached a steady state after co-administration supported the assertion that this constituted appropriate evaluation of the effects of drug–drug interactions, based on referable guidelines [14, 15].

Although co-administration with oxaprozin prolonged the *T*_1/2_ and increased AUC_0–inf_ of dotinurad, the 90% CIs for the geometric mean ratios of all PK parameters of dotinurad in plasma were within the standard range (0.8–1.25) specified in the guidelines for drug–drug interactions [14, 15]. These findings demonstrate that co-administration with oxaprozin had no clinical effect on the PK of dotinurad in plasma.

In comparison with administration of dotinurad alone, the urinary excretion rate of glucuronate conjugates of dotinurad decreased by 34.3% when co-administered with oxaprozin. This was possibly caused by inhibition of glucuronidation or alteration in renal excretory capacity by oxaprozin. The estimated glomerular filtration rates (eGFRs) (mean ± SD) were 100.9 ± 13.0 mL/min/1.73 m^2^ (*n* = 12), 24 h after administration of dotinurad alone; and 93.3 ± 13.0 mL/min/1.73 m^2^ (*n* = 11), 24 h after co-administration with oxaprozin, showing no meaningful change after co-administration. Based on these findings, we estimated that the decreased urinary excretion rate of dotinurad’s glucuronate conjugates after oxaprozin dosing was attributable not to a decline in renal excretion but to inhibition of glucuronidation by oxaprozin. Given that 38.6% of the glucuronate conjugates was excreted in urine after administration of dotinurad alone, the proportion of glucuronidation inhibited by co-administered oxaprozin in terms of the entire metabolism of dotinurad was calculated to be 13.2%. Assuming absence of compensatory enhancement of other metabolic pathways, dotinurad exposure was estimated to increase by approximately 13% when co-administered with oxaprozin, and in fact, the geometric mean ratio of AUC_0–inf_ showed a 16.5% increase (Table 2). Although oxaprozin caused an approximately 34% decrease in the urinary excretion of glucuronate conjugates of dotinurad by inhibition of UGT, it is considered that this effect on the overall PK profile of dotinurad is insignificant because of its proportion with respect to total metabolism.

The finding that co-administration with oxaprozin increased unbound fraction of dotinurad in plasma at the time of *C*_max_ indicated a possibility that clearance (CL) of dotinurad would increase. However, the excretion of sulfate conjugates of dotinurad after co-administration with oxaprozin did not differ from that of dotinurad alone, suggesting that increased unbound fraction of dotinurad in plasma had a negligible effect on dotinurad metabolism.

Co-administration with oxaprozin increased unbound fraction in plasma of dotinurad and slightly increased AUC_0–inf_ due to decreased CL. These changes could augment the pharmacodynamics of dotinurad. However, other clinical studies have confirmed that the pharmacodynamics of dotinurad becomes saturated at ≥ 5 mg, and there seems to be no possibility that PK changes due to co-administration with oxaprozin observed in this study would augment the effect of dotinurad up to a dose of 4 mg [16, 17].

In total, three AEs were reported in two subjects during the study. None of these constituted an adverse drug reaction. Peritonsillar abscess, a serious AE, was caused by infection and its causal relationship to the study drug was ruled out by the investigator. Following co-administration with dotinurad, no subjects experienced the occurrence or worsening of any adverse drug reactions resulting from oxaprozin (e.g., gastrointestinal disorder) that might develop during repeated dosing of oxaprozin.

Taken all these findings together, we conclude that concomitant use of oxaprozin and dotinurad produces no clinically meaningful drug–drug interactions in terms of their PK profiles and safety.

## Conclusions

The results of our study in healthy adult males demonstrated a prolongation of T_1/2_ and an increase in AUC_0–inf_ of dotinurad in plasma due to the inhibitory effect of oxaprozin on the glucuronidation of dotinurad. However, these effects are within the standard range specified in the guidelines for drug–drug interactions and we conclude that there is no clinically meaningful difference. Administration of dotinurad alone and co-administration with oxaprozin were similarly safe without generating any clinically significant events.
